# Efficacy of bevacizumab combined with erlotinib for advanced hepatocellular carcinoma: a single-arm meta-analysis based on prospective studies

**DOI:** 10.1186/s12885-019-5487-6

**Published:** 2019-03-28

**Authors:** Liyun He, Huan Deng, Jun Lei, Fengming Yi, Jine Li, Xiu De Fan, Yiping Wei, Jianjun Xu, Wenxiong Zhang

**Affiliations:** 1 Department of Thoracic Surgery, The Second Affiliated Hospital of Nanchang University, 1 Minde Rd, Nanchang, 330006 Jiangxi Province China; 2 Jiangxi Medical College, Nanchang University, Nanchang, 330006 China; 3 Department of Hepatobiliary Surgery, The Second Affiliated Hospital of Nanchang University, Nanchang, 330006 China; 4 Department of Oncology, The Second Affiliated Hospital of Nanchang University, Nanchang, 330006 China; 5 Department of Infectious Diseases, The First Affiliated Hospital of Xi’an Jiaotong University, Xi’an, 710061 China

**Keywords:** Meta-analysis, Bevacizumab, Erlotinib, Hepatocellular carcinoma, Sorafenib

## Abstract

**Background:**

The efficacy of bevacizumab combined with erlotinib (B + E) for the treatment of advanced hepatocellular carcinoma, especially for sorafenib-refractory patients, has been observed and evaluated in several trials. We conducted this single arm meta-analysis to generally assess the benefit and risk with B + E for advanced hepatocellular carcinoma.

**Methods:**

The PubMed, Cochrane Library, Embase, ScienceDirect, Web of Science and Scopus databases were searched for related studies. The main outcomes were objective response rate (ORR), disease control rate (DCR), overall survival (OS), progression-free survival (PFS) and adverse effects (AEs).

**Results:**

Eight phase II clinical trials including 342 hepatocellular carcinoma patients were analyzed. The pooled ORR was 12.6% (95% CI: 6.3–19.0%), and the pooled DCR was 54.5% (95% CI: 48.9–66.8%). The 16-week PFS rate was 50.2% (95% CI: 38.2–62.2%). The 6- and 12-month OS rates were 77.8% (95% CI: 71.3–84.2%) and 44.9% (95% CI: 36.8–53.0%). The main grade 3–4 AEs were fatigue (11.9%), diarrhea (9.0%), hypertension (6.7%), acne (5.8%) and hemorrhage (5.3%). The only RCT showed that the B + E regimen had a consistent response and equable median OS but fewer toxicities (grade 3–4 AEs: 19% vs. 27%) than sorafenib. Subgroup analysis showed that as a second-line treatment, the B + E regimen had substantial value with a favorable PFS-16w (*P* = 0.012), OS-12 m (*P* = 0.048) and a favorable tendency of ORR (*P* = 0.089), but obvious toxicities in the second-line setting could not be neglected.

**Conclusion:**

Bevacizumab combined with erlotinib is effective for treating hepatocellular carcinoma patients, especially sorafenib-refractory patients. More well-designed and large-scale RCTs are warranted to prove our findings.

**Electronic supplementary material:**

The online version of this article (10.1186/s12885-019-5487-6) contains supplementary material, which is available to authorized users.

## Background

Hepatocellular carcinoma (HCC), classified as the fifth most frequent malignant neoplasm worldwide, is the third leading cause of cancer-related death globally [1]. Alarmingly, the incidence rate of HCC is rising both in Europe and worldwide [2]. Clinically, patients with HCC are frequently initially diagnosed at the late stages, at which point the disease has progressed, accompanied by deteriorated outcomes, advanced disease or distant metastasis, and are generally not eligible for curative therapies; they are confined to systemic therapy [3].

Recognized as the most chemo-resistant tumor type, advanced HCC had no systemic drug recommendation until 2007. Currently, sorafenib, as a multi-kinase inhibitor that impedes tumor cell proliferation and angiogenesis, is the most common-used antineoplastic agent approved as a first-line systematic treatment for patients with advanced or metastatic HCC [4]. However, some clinical trials have depicted unsatisfactory results of sorafenib with limited efficacy and obvious toxicities for certain patients [5]. With HCC being classified as an intractable tumor abundant in angiogenesis, agents targeting angiogenesis have been actively studied but have failed [6, 7]. Although most recently, another multi-kinase inhibitor –lenvatinib, was newly approved by FDA for first-line treatment of patients with unresectable HCC, which shared the approximately similar functional way with sorafenib. Thus, lenvatinib might not work very well on sorafenib-refractory patients. The harsh problems that patients might bear the intolerance and resistance to sorafenib urgently require settlement. Although nivolumab is currently approved for second line for patients intolerant to sorafenib, the post-challenges and opportunistic risks for second-lined therapy strategies for patients with advanced HCC still can’t be ignored.

HCC patients manifest conspicuously elevated levels of vascular endothelial growth factor (VEGF) expression and frequently show increased co-expression of TGF-alpha and EGFR [8], leading to active and massive cell proliferation because TGF-alpha, epidermal growth factor receptor (EGFR) and its ligand EGF play important roles in cell proliferation [9]. Erlotinib, a tyrosine kinase inhibitor that down-regulates VEGF, was reported to be efficacious in advanced HCC [10, 11]. Similarly, bevacizumab, a VEGF inhibitor, manifested impressive values (PFS ranging from 5.3 months to 9.0 months, OS ranging from 5.9 to 13.7 months, and disease control rate [DCR] ranging from 51.1 to 76.9%) in the treatment of advanced HCC [12, 13]. Because the two agents act on targets of different but equally important pathways in hepatocarcinogenesis, the combination of bevacizumab plus erlotinib (B + E) has been attempted in several clinical trials [14]. Most likely due to an additive effect on angiogenesis provided by erlotinib, greater benefits were observed in the treatment of advanced HCC. Preclinical trials in tumors, including HCC, have demonstrated a greater efficacy of the combination of B + E than that of either agent alone [15]. Bevacizumab combined with erlotinib presented great potential and advantages in the treatment of patients with advanced HCC, especially for those with intolerance to sorafenib. The combination of B + E could be a promising second-line treatment after sorafenib for patients with advanced HCC.

Thus, the present study aimed to analyze the efficacy of B + E for advanced HCC, especially in which treatment with sorafenib fails.

## Methods

This meta-analysis was consistent with Preferred Reporting Items for Systematic Review and Meta-analysis (PRISMA) (Additional file 1: Table S1) [16].

### Search strategy

**Fig. 1:**
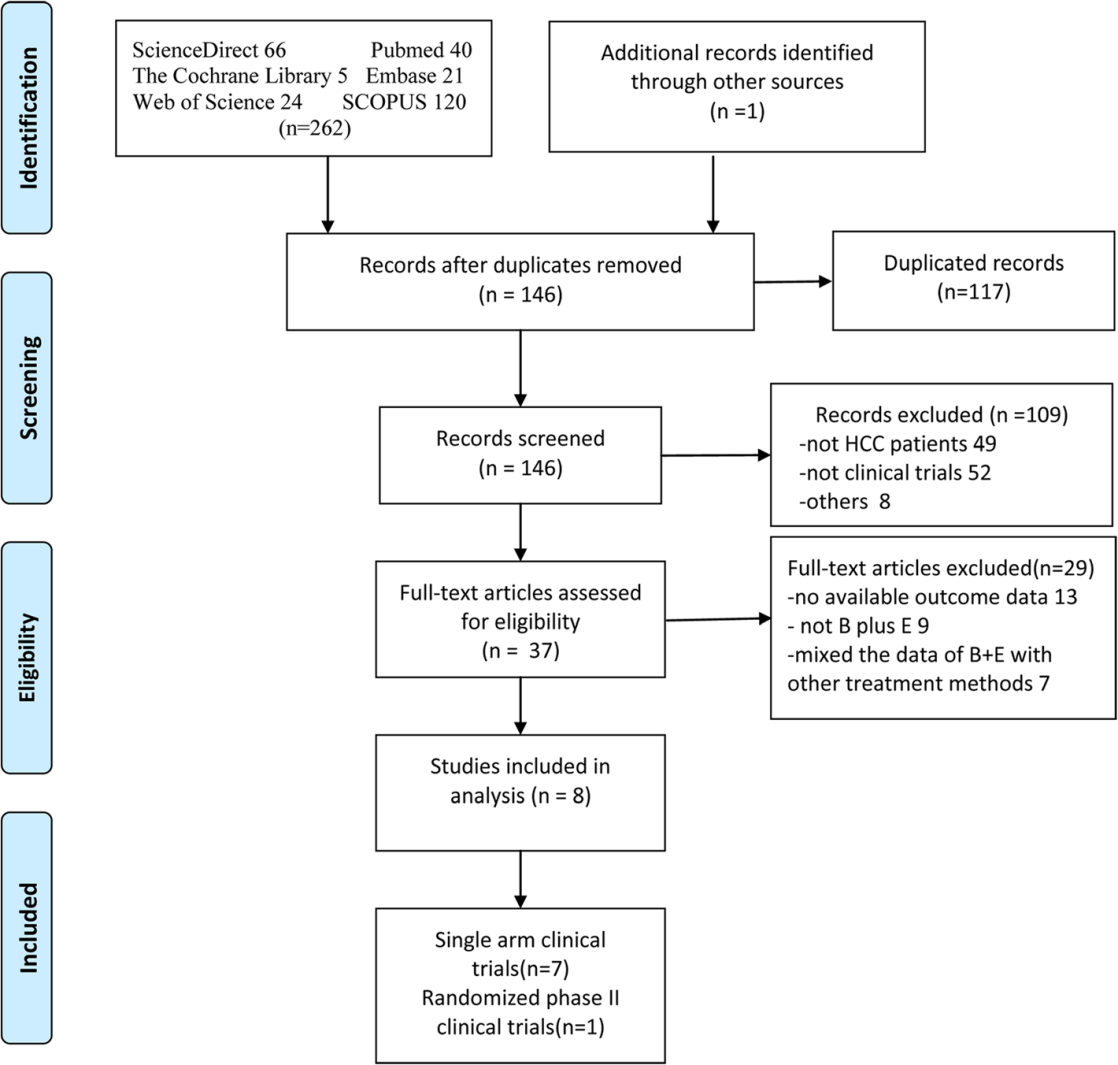
Flow chart of the included studies

We comprehensively retrieved literatures from the PubMed, Cochrane Library, Embase, ScienceDirect, Web of Science and SCOPUS databases from January 1, 2009 to July 5, 2018. The primary search terms were as follows: “Hepatocellular Carcinoma, liver cell carcinoma, liver cancer”, “Bevacizumab, Avastin” and “Erlotinib, erlotinib HCl, OSI-774, Tarceva”. The integrated searches used for PubMed were as follows:(((((((“Hepatocellular Carcinoma” [All Fields] OR “liver cell carcinoma” [All Fields]) OR “liver cancer” [All Fields]) AND (“bevacizumab” [MeSH Terms] OR “bevacizumab” [All Fields])) OR “avastin” [All Fields])) AND (“erlotinib hydrochloride” [MeSH Terms] OR (“erlotinib” [All Fields] OR “erlotinib HCl” [All Fields]) OR “Osi 774” [All Fields])) OR “tarceva” [All Fields]) AND Clinical Trial [Ptyp]. In addition, the references of the acquired literature were reviewed to determine whether any other qualified studies were missed. The search strategy flow chart is exhibited in Fig. 1.

### Selection criteria

The inclusion criteria were as follows: (1) studies on adult patients with advanced HCC; (2) B + E was the main treatment; (3) no limits of language, race, region, sex or pretreatment; (4) the primary outcomes of ORR, overall survival rate at 6 or 12 months (OS-12 m and OS-6 m), progression-free survival rate at16 weeks (PFS-16w), any-grade adverse effects (any-grade AEs), and grade 3–4 adverse effects (grade 3–4 AEs) were available directly or indirectly; and (5) prospective studies.

The exclusion criteria were as follows: (1) letters, meta-analyses, reviews, and animal trials; (2) bevacizumab or erlotinib in combination with other drugs; (3) bevacizumab or erlotinib was not the main treatment for advanced HCC patients; and (4) studies without usable data.

### Data extraction

The relevant data were extracted by two investigators from eligible studies, and the following variables were collected: (1) name of first author, publication year, region, age of patients, number of patients in each study, gender, distribution of race; (2) liver function (Child-Pugh classification), the Cancer of the Liver Italian Program (CLIP) [17, 18] and Eastern Cooperative Oncology Group (ECOG) performance statuses, background liver disease, number of patients receiving prior treatment with sorafenib, line of treatment; (3) objective response rate (ORR), DCR, OS-12 m and OS-6 m, PFS-16w, any-grade AEs, and grade 3–4 AEs; and (4) partial response rate (PR), stable disease rate (SD), progressive disease rate (PD), OS, PFS, median time to progression (TTP). All obtained information and original data were put in standardized collection tables and checked by a third investigator. Disagreements were settled by consensus after discussion with a third investigator.

### Quality assessment

The Cochrane risk of bias tool was applied for methodological quality judgment of the only randomized controlled study [19]. Nevertheless, the remaining seven single-arm literature sources did not have textbook-quality guidelines-i.e., large heterogeneity might exist for these single-arm literature sources [20].

### Statistical analysis

All statistical analyses were performed using Stata statistical software version 14.0, and *P* < 0.05 was considered statistically significant. The total clinical setting rates of the primary outcome, number of patients in each study and corresponding standard errors calculated by Stata were then used to assess the efficacy of the combination of B + E. The final pooled effect sizes were modified by abandoning studies with large variability based on sensitive analysis results. Heterogeneity between studies was evaluated by the Cochran Q chi-square test and *I*^*2*^ statistics, and *P* < 0.10 indicated apparent heterogeneity. Heterogeneity was classified as low (*I*^*2*^ < 50%) or high (*I*^*2*^ > 50%). Considering the absence of corresponding single-arm trials that might exist subjectively, random-effects models were applied for all pooled effect sizes. Subgroup analyses were performed according to the ECOG, prior systematic therapy, region, line of treatment, and liver functions but only for ORR, OS-12 m and grade 3–4 AEs because of limits to accessible data. The significance of the overall results was judged by the *Z*-test, with *P* < 0.05 indicating statistical significance. Egger’s test was used to evaluate latent publication bias among the primary outcomes.

## Results

### Study identification and characteristics

From the databases mentioned previously, 262 studies were obtained, and one study was obtained from another source; the entire process is exhibited in Fig. 1. Initially, 117 duplicated articles were removed, and the remaining 146 studies were excluded according to the exclusion criteria. Finally, the remaining 8 articles including 342 hepatocellular carcinoma patients were eligible for the present meta-analysis, comprising seven single-arm trials [21–27] and one randomized controlled trial [28]. Additionally, the primary characteristics of the eligible studies are presented in Table 1. The patients of these studies came from the United States, Egypt, China, Hong Kong and Taiwan and comprised mainly four races: Caucasian, African American, Asian and Hispanic (Additional file 2: Table S2). All participants were confined to Child-Pugh classes A or B and ECOG performance status of 0–1 or 0–2, and most were at Barcelona Clinic Liver Cancer (BCLC) stage C. A few patients had background liver disease, which might have decreased the effectiveness of B + E treatment. Most of the included trials adopted the treatment strategies that patients received 10 mg/kg bevacizumab every 14 days and 150 mg of erlotinib administered orally daily. Furthermore, the main outcomes are depicted in Table 2.

**Table 1:** Characteristics of the included prospective cohort studies

Study	Region	N	Age	Male/female	Schedule	Child-Pugh	CLIP (0/1/2/3/4)	Prior therapy	ECOG (0/1/2)	BCLC (A/B/C)	Liver disease	Line of therapy
Melanie 2018 [28]	USA	90	61	NA	Arm1: S(400 mg, Q2D, oral) Arm2: B (10 mg/kg, Q2w) + E (150 mg/d, oral)	S:A88%;B12% B + E:A83%;B17%	S:4/10/17/9/3 B + E:7/10/16/8/6	NA	S:17/25/1 B + E: 15/32/0	S: A9%B26%C65% B + E: A2%B30%C68%	NA	First
Kaseb 2016 [24]	Egypt	44	63	33/11	B(10 mg/kg, Q2w) + E(150 mg/d, oral)	A98%, B2%	5/17/12/7/0	Sorafenib	15/29/0	A5%B2%C93%	HBV8; HCV13 Alcoholism10 Cirrhosis19	Second
Govindarajan 2013 [21]	USA	21	60	13/8	B(15 mg/kg, Q3w) + E(150 mg/d, oral)	A85.7%, B14.3%	NA	NA	NA	A0%B23.8% C76.2%	HBV1; HCV6 HCV + HBV2	First
Hsu 2013 [22]	Taiwan	51	58	44/7	B(5 mg/kg, Q2w) + E (150 mg/d, oral)	A98%, B2%	15/8/11/12/5	NA	30/20/1	A0% B11.7% C88.3%	HBV42;HCV4 HBV + HCV3	First
Philip 2012 [25]	USA	27	60	20/7	B(10 mg/kg, Q2w) + E(150 mg/d, oral)	A74%, B26%	NA	Sorafenib	16/11/0	NA	HBV1; HCV8 Alcoholism2; Cirrhosis6	Second
Yau 2012 [27]	China	10	47	7/3	B(10 mg/kg, Q2w) + E(150 mg/d, oral)	A100%	NA	Sorafenib	0/10/0	A0% B10%, C90%	HBV10 Cirrhosis10	Second
Kaseb 2012 [23]	USA	59	64	13/46	B(10 mg/kg, Q2w) + E(150 mg/d, oral)	A86% B14%	3/14/13/22/7	Sorafenib(7/59)	NA	A3.4%B20.3% C76.3%	HBV10; HCV17 Alcoholism 27	NA
Melanie 2009 [26]	USA	40	64	31/9	B(10 mg/kg, Q2w) + E(150 mg/d, oral)	A87.5%, B12.5%	2/11/6/16/5	Sorafenib(8/40)	19/20/1	A5%B30% C65%	HBV6; HCV10 Alcoholism17 Cirrhosis27	NA

Note: *B* bevacizumab, *E* erlotinib, *S* sorafenib, *NA* not available

**Table 2:** Summary of the antitumor effects

Study	N	ORR	DCR	CR	PR	SD	PD	PFS (mon) Median (95% CI)	TTP (mon) (95%CI)	PFS-16w	OS (mon) Median (95% CI)	OS-12 m	OS-6 m	Grade3–4 AEs
Melanie 2018 [28]	90	15%	NA	NA	NA	NA	NA	4.37 (2.99–7.36)	NA	NA	8.6 (7.0–13.9)	37%	71.3%	19%
Kaseb 2016 [24]	44	9%	50%	0	9%	41%	9%	3.9 (2.0–8.3)	3.9 (2.0–8.3)	43%	9.9 (8.3–15.5)	NA	NA	NA
Govindarajan 2013 [21]	21	0	NA	NA	NA	NA	NA	2.57 (2.13–4.20)	2.57 (2.13–4.2)	28%(27w)	8.33 (5.73–13.97)	41.6%	68.7%	NA
Hsu 2013 [22]	51	6%	53%	NA	6%	47%	45%	2.9 (1.3–4.4)	2.9 (1.3–4.4)	35.3%	10.7 (6.2–15.2)	47.5%	69.1%	43.1%
Philip 2012 [25]	27	4%	52%	NA	4%	48%	48%;	3.0 (1.8–7.1)	3.0 (1.8–7.1)	NA	9.5 (7.1–17.1)	40.4%	74.4%	58%
Yau 2012 [27]	10	0	0	NA	0	0	0	1.51 (1.08–1.74)	1.81 (1.08–1.74)	NA	4.37 (1.08–11.66)	12.4%	38.1%	20%
Kaseb 2012 [23]	59	24%	80%	NA	24%	56%	10%	7.2 (5.6–8.3)	NA	64%	13.7 (9.6–19.7)	57.1%	83.6%	NA
Melanie 2009 [26]	40	25%	62.5%	NA	25%	37.5%	NR	9.0 (6.0–10.4)	NA	62.5%	15.7 (11–18)	63.1%	83.0%	22.5%

Note: *ORR* objective response rate, *DCR* disease control rate, *CR* complete response rate, *PR* partial response rate, *SD* stable disease rate, *PD* progressive disease rate, *PFS* progression-free survival, *OS* overall survival, *TTP* median time to progression, *AEs* adverse effects, *NA* not available

### Quality assessment

Using the Cochrane risk of bias tool, the only randomized controlled study generated random sequences, provided complete outcome data and was free of other bias but did not present allocation concealment and blinding methods. However, we included single-arm studies from top journals, ensuring their high quality and integrity.

### Tumor response

Both ORR and DCR were analyzed, whereas CR was not analyzed due to the paucity of data available. The ORR data were available for analysis from 8 trials including 342 patients. The pooled ORR was 12.6% (95% CI: 6.3–19.0%) as determined by the random-effects model (heterogeneity analysis: *I*^*2*^ = 70.0%, *P* = 0.005) (Additional file 3: Table S3). The results of sensitivity analysis suggested that arbitrarily omitting any one study didn’t obviously decrease the ORR heterogeneity (Additional file 4: Fig. S1).

DCR, ranging from 50 to 80%, was available for analysis in 5 trials with 221 participants [21–25]. The pooled DCR was 60.3% (95% CI: 47.3–73.3%) as determined by the random-effects model (heterogeneity analysis: *I*^*2*^ = 76.4%, *P* = 0.002). After omitting one study with large heterogeneity based on the results of sensitivity analysis, the pooled ORR was54.5% (95% CI: 48.9–66.8%) with extremely low heterogeneity (*I*^*2*^ = 0, *P* = 0.668) (Additional file 5: Table S4, Additional file 6: Fig. S2).

### PFS

Five studies reported the PFS at 16 weeks (PFS-16w), and one study [20] described the PFS at 27 weeks (PFS-27w) (28%). The pooled PFS was 46.9% (95% CI: 35.1–58.8%) with high heterogeneity (*I*^*2*^ = 72.9%, *P* = 0.002). The pooled PFS-16w was 50.2% (95% CI: 38.2–62.2%) with high heterogeneity (*I*^*2*^ = 70.6%, *P* = 0.0009) (Additional file 7: Table S5). Sensitivity analysis demonstrated that the PFS rate of this meta-analysis was considered stable, and arbitrarily omitting any one study didn’t decrease the heterogeneity (Additional file 8: Fig. S3).

### OS

The overall survival rates at 6 months (OS-6 m) and 12 months (OS-12 m) were analyzed respectively.

The OS-6 m rates, ranging from 38.1 to 83.6%, were available for analysis in 208 patients from 6 trials [20–22, 24–26]. The overall OS-6 m rate was 74.0% (95% CI: 64.8–83.2%) with high heterogeneity (*I*^*2*^ = 56.8%, *P* = 0.041). After omitting one study with apparent heterogeneity and sensitivity after sensitivity analysis [27], the modified overall OS-6 m rate was 77.8% (95% CI: 71.3–84.2%) with low heterogeneity (*I*^*2*^ = 19.5%, *P* = 0.290) (Additional file 9: Table S6, Additional file 10: Figure S4A).

Analyzed from the data of 298 patients in 7 trials [20–22, 24–27], the OS-12 m rate ranged from 12.4 to 57.1%. The pooled OS-12 m rate was 43.7% (95% CI: 32.9–54.6%) with high heterogeneity (*I*^*2*^ = 72.6%, *P* = 0.001). After omitting two studies with substantial heterogeneity and sensitivity after sensitive analysis [27, 28], the pooled OS-12 m rate was 44.9% (95% CI: 36.8–53.0%) with a lower heterogeneity (*I*^*2*^ = 37.9%, *P* = 0.169) (Additional file 11: Table S7, Additional file 10: Figure S4B).

### Toxicity

The common toxicities accompanying any-grade AEs in treatment with the B + E regimen were diarrhea (54.4%), acne (51.1%), fatigue (46.5%), hemorrhage (36.8%), and anorexia (34.2%). Grade 3–4 toxicities, including fatigue (11.9%), diarrhea (9.0%), hypertension (6.7%), acne (5.8%) and hemorrhage (5.3%). Additional file 12: Table S8 demonstrates the relatively common toxicities in detail. Relatively common toxicities of any grade and grade 3–4 in each study were presented in Additional file 13: Table S9 and Additional file 14: Table S10.

### Subgroup analysis

To explore the treatment effect of the B + E regimen across various subgroups, subgroup analysis was performed using the following classification variables: ECOG performance status (0–1, 0–2), pre-schedule (prior systematic therapy, no systematic therapy), line of treatment (first line, second line) region (United States, non-United States), and liver function (Child-Pugh classes A and B, Child-Pugh class A). Because of the limits of unavailable data, only the objective response rate (ORR) and overall survival at 12 months (OS-12 m) were involved in subgroup analysis. The final results are on displayed in Table 3. The stratification of ECOG and pre-schedule did not show obvious differences in ORR and OS-12 m, while significant differences were found in grade 3–4 AEs (*P* < 0.05). The United States populations had a higher ORR than the non-United States populations (16.1% vs. 7.1%, *P* = 0.014), while no significant differences in OS-12 m and grade 3–4 AEs were noted. A significant difference was noted between the Child-Pugh class A/B and Child-Pugh class A groups in OS-12 m and grade 3–4 AEs, whereas no obvious difference was found in the ORR. Regarding heterogeneity, the pre-schedule, region and liver function were most likely the main sources of heterogeneity.

**Table 3:** Subgroup analysis of the objective response rate (ORR), overall survival rate at 12 months (OS-12 m) and grade 3–4 adverse effects (grade 3–4 AEs) of B + E in patients with advanced HCC

	ORR				OS-12 m			
Group	N	ES (95% CI)	P Values	*I*^*2*^ (%)	N	ES (95% CI)	P Values	*I*^*2*^ (%)
Total	342	0.126 (0.063,0.190)	–	70.0%	298	0.437 (0.329,0.546)	–	72.6%
ECOG								
0–1	176	0.117 (0.009,0.224)	0.956	77.6%	176	0.376(.121,0.631)	0.701	85.1%
0–2	166	0.140 (0.044,0.236)		73.3%	122	0.470 (0.351,0.589)		63.9%
Pre-schedule								
Prior systematic therapy	180	0.145 (0.044,0.247)	0.128	77.3%	136	0.443 (0.243, 0.644)	o.456	83.5%
No systematic therapy	162	0.103 (0.015,0.191)		67.9%	162	0.408 (0.332, 0.483)		0.0%
Line of treatment								
First line	162	0.103 (0.015,0.191)	0.089	68.9%	162	0.408 (0.332, 0.483)	0.048	0.0%
Second line	180	0.145 (0.044,0.247)		77.3%	37	0.267(−0.007,0.542)		83.5%
Region								
America	237	0.161 (0.068,0.258)	0.014	76.7%	237	0.481 (0.369,0.592)	0.517	65.2%
Not America	105	0.071 (0.020,0.123)		0.0%	61	0.308(−0.036,0.652)		87.2%
Liver functions								
Child-Pugh class A/B	147	0.170 (0.019,0.321)	0.224	84.2%	147	0.524 (0.418,0.630)	< 0.001	40.5%
Child-Pugh class A	195	0.098 (0.044,0.153)		38.4%	151	0.341 (0.179,0.503)		74.4%

Note: Liver functions: the subgroup of Child-Pugh class A and B refers patients enrolled with liver function of Child-Pugh class B excess 10%, otherwise the subgroup of Child-Pugh class A. *ES* Effect size, *ORR* objective response rate, *OS* overall survival, *AEs* adverse effects, *B + E* bevacizumab combined with erlotinib

A comparison of the primary outcomes between the first and second lines of treatment is shown in Table 4. At the second-line settings, the PFS-16w was obviously higher than that in first-line treatment (54.5% vs. 35.3%; *P* = 0.012). The difference in OS-12 m was also significant (*P* = 0.048), with higher rates being observed with second-line treatment than with first-line treatment (44.3% vs. 40.8%). However, no significant differences were detected in DCR, ORR or OS-6 m. But there was a trend favoring second-line treatment in ORR (0.145 vs. 0.103). Additionally, second-line treatment revealed higher grade 3–4 AEs (67.0% vs. 0.512%; *P* = 0.005), which could not be neglected.

**Table 4:** Comparison of the primary outcomes between the first- and second-line treatments in the included studies

	ORR		DCR		PFS-16w			OS-12 m	OS-6 m	
	n	95% CI	n	95% C	n	95% C	n	95% C	n	95% C
Overall	342	0.126 (0.063,0.190)	221	0.603 (0.473,0.733)	221	0.502 (0.382,0.622)	298	0.437 (0.329,0.546)	298	0.739 (0.664,0.813)
First line	162	0.103 (0.015,0.191)	51	0.530 (0.393,0.667)	51	0.353 (0.222,0.484)	162	0.408 (0.332,0.483)	162	0.703 (0.633,0.773)
Second line	180	0.145 (0.044,0.247)	170	0.620 (0.465,0.775)	170	0.545 (0.433,0.656)	136	0.443 (0.243,0.644)	136	0.757 (0.632,0.882)
*P* values	0.089		0.174		0.012		0.048		0.050	

Note: Grade 3–4 AEs was obtained by adding all the adverse events in grade 3–4 together. *ORR* objective response rate, *DCR* disease control rate, *PFS-16w* progression-free survival rate at 16 weeks, *OS-12 m* overall survival rate at 12 months; *OS-6 m* overall survival rate at 6 months

### Publication bias diagnosis

**Fig. 2:**
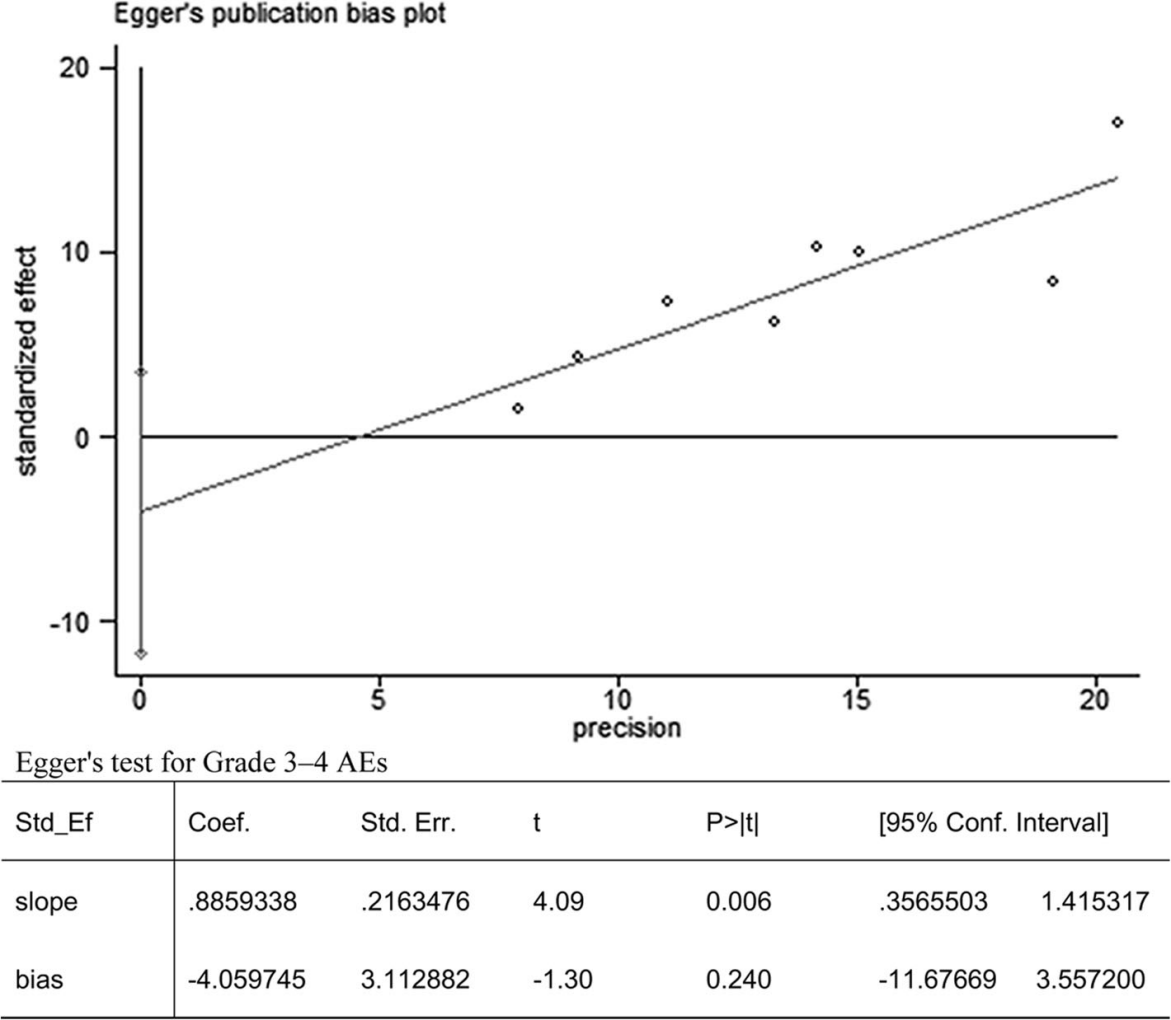
Results of the Egger’s test for grade 3–4 AEs

No obvious publication bias existed as determined by analysis with Egger’s test (*P* = 0.240) based on the analysis of grade 3–4 AEs (Fig. 2).

## Discussion

This was the first far-reaching, single-arm meta-analysis to evaluate the efficacy of B + E for patients with advanced or metastatic hepatocellular carcinoma (HCC). Despite the inconspicuous ORR, a clear clinical benefit, favorable median OS and OS-12 m, and mild toxicity in advanced HCC were achieved with B + E treatment. The combination of B + E showed moderate activity and tolerable adverse events in the treatment for advanced HCC. Remarkably, the only randomized, open-label multi-institution study favored the B + E combined regimen, showing a better toxicity profile and response rate for advanced HCC patients than that achieved with the sorafenib-alone regimen, while the OS data were approximately paralleled [28]. With moderate efficacy, B + E could be a promising second-line treatment regimen for HCC patients with advanced disease or metastases when sorafenib fails. This treatment regimen is meaningful and bears substantial value in clinical practice, especially in patients refractory to sorafenib. Sorafenib, the only drug approved for the systematic treatment of patients with advanced or metastatic HCC, was reported to show moderate, limited efficacy in some cases, with patients who were refractory to sorafenib not being uncommon. The B + E regimen could exert a favorable impact on these patients.

This study demonstrated that the PFS-16w and OS-12 m rates for advanced patients treated with the B + E regimen were 50.2% (95% CI: 38.2–62.2%) and 44.9% (95% CI: 36.8–53.0%), respectively, indicating an elevated survival effect. In addition, the clinical benefits manifested by the overall DCR of 54.5% (95% CI: 48.9–66.8%) and ORR of 12.6% (95% CI: 6.3–19.0%) indicated advantages over conventional chemotherapy that were reported with low response rates and lacked a beneficial impact on the treatment of patients with advanced HCC [28]. The toxicities were tolerable and manageable, whereas cytotoxic chemotherapy universally induced significant toxicity. Additionally, inadequate evidence existed for chemotherapy as a second-line treatment for patients with advanced HCC [29]. Regorafenib, a second-line systematic drug approved in 2017 for advanced HCC patients who progressed on or after sorafenib, showed approximately similar benefit rates and activities to the B + E regimen [30]. Nevertheless, patients administered regorafenib manifested universal adverse events, with grade 3–4 treatment-related AEs occurring most frequently. Additionally, seven deaths occurred that were largely considered to be due to treatment-related AEs. Regarding safety, the B + E regimen may have an obvious advantage. Newly reported trials demonstrated that approximately only 30% of patients who progressed on sorafenib were eligible for treatment with regorafenib, whereas patients with hypoalbuminemia at sorafenib initiation were scarcely eligible [31]. In addition, another important usage limitation was that regorafenib, with a similar mechanism to that of sorafenib, could not function in patients who were not tolerant to sorafenib [2]. Possessing substantial potential, the combination of B + E might be helpful for patients who are ineligible for regorafenib in future studies. Additionally, other systematic drugs, such as everolimus, brivanib, and ramucirumab, that were evaluated at the second-line treatment setting showed no clear survival benefits [4].

Furthermore, the only randomized controlled trial included in our study displayed a similar median OS (8.6 months) and generally consistent ORR (15 and 9%) with the B + E regimen for patients with advanced HCC compared with sorafenib, but the B + E regimen was associated with fewer AEs than sorafenib (19% vs. 27%) based on competing risk analysis [27]. Thus, the foundation is set for the B + E regimen to be used as a second-line treatment after sorafenib.

Although three clinical trials evaluated the efficacy of bevacizumab plus erlotinib for advanced HCC patients as a first-line treatment [21, 22, 28], the primary outcomes of first-line treatment were generally inferior to those of second-line treatment, as shown in Table 4. Compared with first-line treatment, the second-line treatment obtained higher PFS-16w (54.5% vs. 35.3%; *P* = 0.012) and significantly OS-12 m (44.3% vs. 40.8%; *P* = 0.048), and the outcomes were closely related to survival benefits. Additionally, a trend of elevated ORR was noted in the second-line settings. However, the obviously higher grade 3–4 AEs in the second-line settings could not be neglected in the usage of the B + E regimens for second-line treatment. Most likely, the prior usage of other drugs could aggravate the adverse events. In general, the second-line treatment demonstrated advantages over the first-line treatment to some extent. Conclusively, considering its moderate benefit response, advantages over first-line treatment and the current clinical status of sorafenib, we tend to use the B + E regimen as a second-line treatment for patients with advanced HCC.

Obviously, six of the trials presented only mild efficacy with some activities of response, and two trials in the United States populations yielded better results with a longer median OS and more favorable ORR. Subgroup analysis suggested that receiving prior systematic therapy and second-line treatments probably exerted a favorable influence to some extent. Additionally, the demographics and geographical location of the study population might be responsible for the differences to some extent. As shown in Table 3, the United States population had higher ORRs than the non-United States population (16.1% vs. 7.1%, respectively; *P* = 0.014). This result may have been due to the fact that HCC in the Asian population generally tended to be accompanied by high chronic hepatitis B virus infection, in contrast to HCC western patients who were more likely to be associated with hepatitis C virus infection and alcoholism [24]. Furthermore, patients with an ECOG 1–2 (*P* = 0.016) who received prior systematic therapy (*P* = 0.001) and had a liver function of Child-Pugh class B excess of 10% (*P* < 0.001) showed elevated 3–4 AEs rates, which were likely partially responsible for the high grade 3–4 AEs observed in the second-line trials.

Several included studies elaborated that the outcomes were closely associated with clinical predictors and some biomarkers [20–22]. It was universally reported that patients with a liver function of Child-Pugh class B had shorter median OS and PFS rates than those with Child-Pugh class B given the same sorafenib treatment [2, 32]. Other clinical predictors were hepatitis B infection, history of alcohol intake, declined baseline hemoglobin levels, elevated baseline α-fetoprotein levels, increased baseline alkaline phosphatase levels, tumor volume outnumbering 50%, ill tumor morphology, existence of lymphatic metastasis, ECOG-PG ≥ 1, and CLIP score > 3 accompanied by an inferior prognosis in HCC [23, 32]. Even gender and age might be slightly relevant, as male gender and younger age had an inferior response to the B + E treatment regimen [22, 23]. Considering the absence of standard strategies for HCC as second-line treatment [2], these observations place substantial importance on the stratification of HCC patients in clinical trials based on clinical predictor biomarker studies to select potential patients most appropriate for the B + E treatment regimen who will receive the best benefit [23]. Furthermore, further examination of specific genes and examination on HCC patients who are more effective with B + E regimen treatment should be done in advanced studies and researches.

Nevertheless, high heterogeneity existed in these included studies. The random-effects model was applied when heterogeneity existed within a group to minimize the bias. Substantial efforts were made to explore the possible sources for heterogeneity, revealing that different regions and lines of treatment, various prior administrations and different liver functions could be the main sources of high heterogeneity. In addition, eligible trials with a mixed inclusion of Child-Pugh classes A and B patients could, to some extent, confound the results in that Child-Pugh class B patients have a shorter OS than Child-Pugh class A patients [32]. Similarly, the inclusion of BCLC stage A patients in some trials might impact the final efficacy of treatment with the B + E regimen and cause confusion. However, the inclusion of BCLC stage A patients in some trials did not obviously affect he OS of the B + E and S arm [27].

Several other limitations existed in this meta-analysis. First, the present study, including only one multi-institution, randomized controlled trial, was slightly limited because the included trials were almost all single-arm phase II clinical trials. Therefore, control arms were lacking, and the highest possible quality could not be ensured. However, the single-arm phase II trial design similarly shows significance in the treatment of advanced HCC because the response to standard therapeutic options remains limited, and this could provide research foundations for further studies in this area. Second, two single-arm trials did not observe any objective response to the B + E regimen for advanced HCC patients who were refractory to sorafenib [33]. However, one study was very small in size, an obvious optional bias that reminded us to regard the results with caution [27], and the other study with no observed objective response presented some survival benefits [21]. Finally, some included studies lacked adequate data. For example, OS-6 m and OS-12 m was not available in Kaseb 2016 [24] and 3/8 studies did not provide finally pooled rates of grade 3–4 AEs.

## Conclusions

Our study suggested that the B + E combined regimen appears to be safe and effective for treating advanced HCC, especially as a second-line treatment after sorafenib. The main adverse events included fatigue, diarrhea, hypertension, acne and hemorrhage. Patients with an ECOG 1–2 who received prior systematic therapy and had a liver function of Child-Pugh class B excess of 10% showed obviously higher grade 3–4 AEs than its counterparts. In second-line settings, the B + E regimen demonstrated a favorable PFS-16w and OS-12 m as well as a favorable tendency in ORR (0.145 vs. 0.103), but obvious toxicities could not be neglected. More high-quality RCTs with large samples are urgently needed to further confirm our results.

## Additional files

Additional file 1: **Table S1.** PRISMA checklist. (DOC 64 kb)

Additional file 2: **Table S2.** Distribution of race in the included studies. (DOCX 13 kb)

Additional file 3: **Table S3.** Pooled objective response rate (ORR) in the included advanced HCC patients. (DOCX 14 kb)

Additional file 4: **Figure S1.** Sensitivity analysis of ORR. (TIF 309 kb)

Additional file 5: **Table S4.** Pooled disease control rate. (DCR) and modified DCR in the included advanced HCC patients. (DOCX 14 kb)

Additional file 6: **Figure S2.** Sensitivity analysis of DCR. (TIF 248 kb)

Additional file 7: **Table S5.** Pooled progression-free survival rate. (PFS) and PFS-16w in the included advanced HCC patients. (DOCX 14 kb)

Additional file 8: **Figure S3.** Sensitivity analysis of FPS (A) and FPS-16w (B). (TIF 14110 kb)

Additional file 9: **Table S6.** Pooled overall survival rate at 6 months (OS-6 m) and modified OS-6 m in the included advanced HCC patients. (DOCX 14 kb)

Additional file 10: **Figure S4.** Sensitivity analysis of OS-6 m (A) and OS-12 m (B). (TIF 14345 kb)

Additional file 11: **Table S7.** Pooled overall survival rate at 12 months (OS-12 m) and modified OS-12 m in the included advanced HCC patients. (DOCX 14 kb)

Additional file 12: **Table S8.** Pooled any-grade adverse effects (any-grade AEs) and grade 3–4 AEs in the included advanced HCC patients. (DOCX 15 kb)

Additional file 13: **Table S9.** Relatively common AEs of any grade in each included study. (DOCX 14 kb)

Additional file 14: **Table S10.** Relatively common AEs of 3–4 grade in each included study. (DOCX 14 kb)

## Abbreviations

AEs: Adverse effects
B + E: Bevacizumab combined with erlotinib
DCR: Disease control rate
EGFR: Epidermal growth factor receptor
HCC: Hepatocellular carcinoma
ORR: Objective response rate
OS: Overall survival
PD: Progressive disease rate
PFS: Progression-free survival
PR: Partial response rate
PRISMA: Preferred Reporting Items for Systematic Review and Meta-analysis
SD: Stable disease rate
TTP: Median time to progression
VEGF: Vascular endothelial growth factor
