# Supplementary material for: Efficacy of bevacizumab combined with erlotinib for advanced hepatocellular carcinoma: a single-arm meta-analysis based on prospective studies
Source: BMC Cancer. 2019 Mar 28;19:276. doi: 10.1186/s12885-019-5487-6 (PMC6437948; doi:10.1186/s12885-019-5487-6)
Supplement: Supplementary file 2 — Table S2. Distribution of race in the included studies. (DOCX 13 kb) [file 12885_2019_5487_MOESM2_ESM.docx]

**Table S2.** **The distribution of race in included studies**

| **Study** | **N** | **White** | **Black** | **Hispanic** | **Asian** | **Unknow** |
| --- | --- | --- | --- | --- | --- | --- |
| Thomas 2018 | 90 | 28(31%) | NA | NA | NA | NA |
| Kaseb 2016 | 44 | 26(59%) | - | 9(20%) | 3(7%) | 6(14%) |
| Govindarajan 2013 | 21 | 18(86%) | 3(14%) | - | - | - |
| Hsu 2013 | 51 | NA | NA | NA | NA | NA |
| Philip 2012 | 27 | 21(78%) | 4(15%) | - | 1(4%) | 1(4%) |
| Yau 2012 | 10 | NA | NA | NA | NA | NA |
| Kaseb 2012 | 59 | 40(68%) | 10(17%) | 6(10%) | 3(5%) | - |
| Thomas 2009 | 40 | 25(62.5%) | 8(20%) | 4(10%) | 3(7.5%) | - |

Note: The distribution of race in Melanie (2018) was white (28) and other (19). NA: not available.
