# Supplementary material for: Efficacy of bevacizumab combined with erlotinib for advanced hepatocellular carcinoma: a single-arm meta-analysis based on prospective studies
Source: BMC Cancer. 2019 Mar 28;19:276. doi: 10.1186/s12885-019-5487-6 (PMC6437948; doi:10.1186/s12885-019-5487-6)
Supplement: Supplementary file 3 — Table S3. Pooled objective response rate (ORR) in the included advanced HCC patients. (DOCX 14 kb) [file 12885_2019_5487_MOESM3_ESM.docx]

**Table S3.** Pooled objective response rate (ORR) in advanced HCC patients included

|  |  | **ORR** | |
| --- | --- | --- | --- |
| **Study** | **N** | **Mean** | **95%CI** |
| Thomas 2018 | 90 | 0.15 | (0.076,0.224) |
| Kaseb 2016 | 44 | 0.09 | (0.005,0.175) |
| Govindarajan 2013 | 21 | 0 | 0 |
| Hsu 2013 | 51 | 0.06 | (-0.005,0.125) |
| Philip 2012 | 27 | 0.04 | (-0.034 ,0.114) |
| Yau 2012 | 10 | 0 | 0 |
| Kaseb 2012 | 59 | 0.24 | (0.131, 0.349) |
| Thomas 2009 | 40 | 0.25 | (0.116,0.384) |
| **Total** | 342 | 0.126 | (0.063,0.190) |
| Overall (*I^2^*=70.0%, P=0.005); Egger’s test (P=0.049) | | | |
