# Supplementary figures and images for: Efficacy of bevacizumab combined with erlotinib for advanced hepatocellular carcinoma: a single-arm meta-analysis based on prospective studies
Source: BMC Cancer. 2019 Mar 28;19:276. doi: 10.1186/s12885-019-5487-6 (PMC6437948; doi:10.1186/s12885-019-5487-6)

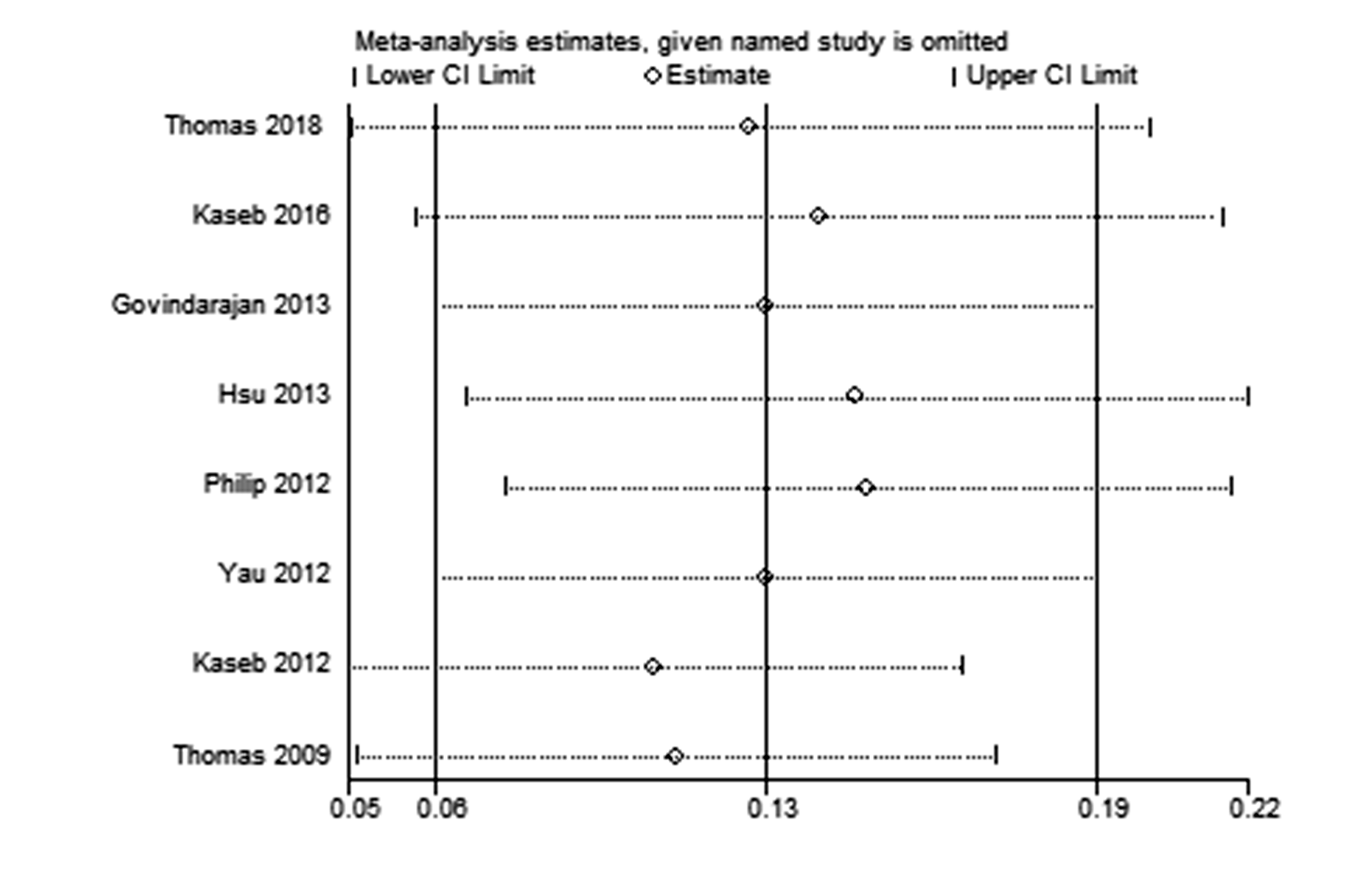

Supplement: Supplementary file 4 — Figure S1. Sensitivity analysis of ORR. (TIF 309 kb) [file 12885_2019_5487_MOESM4_ESM.tif]

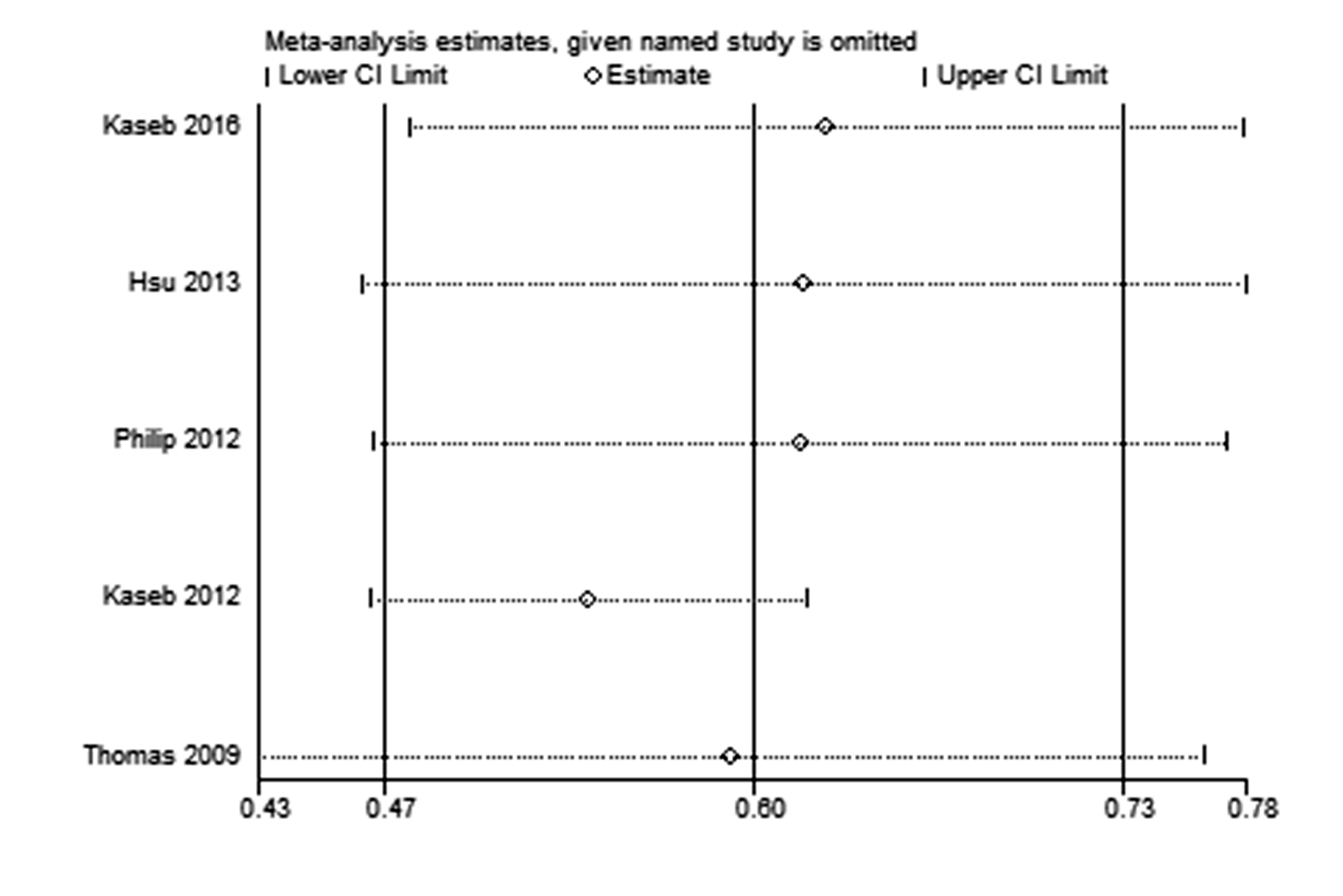

Supplement: Supplementary file 6 — Figure S2. Sensitivity analysis of DCR. (TIF 248 kb) [file 12885_2019_5487_MOESM6_ESM.tif]

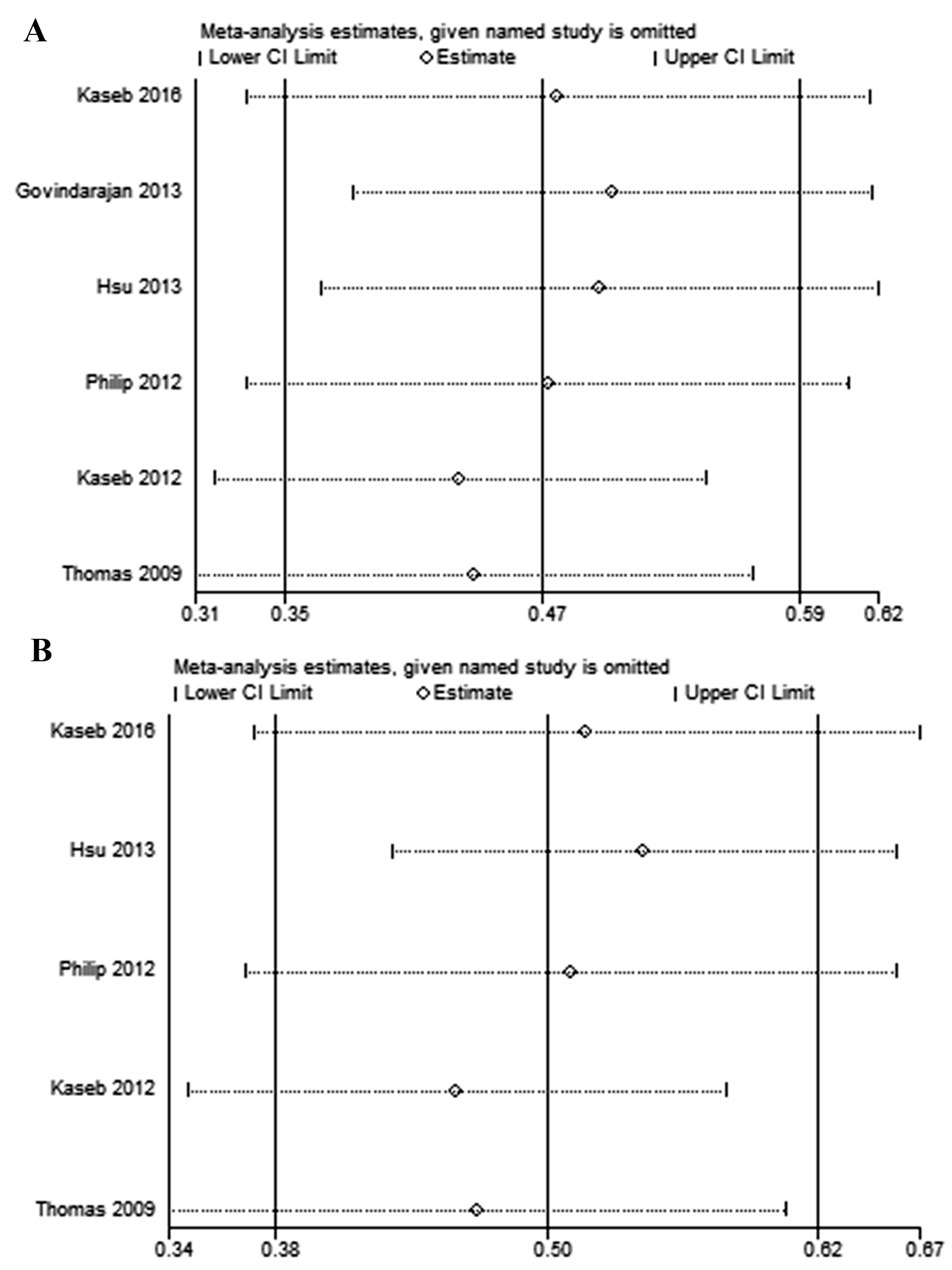

Supplement: Supplementary file 8 — Figure S3. Sensitivity analysis of FPS (A) and FPS-16w (B). (TIF 14110 kb) [file 12885_2019_5487_MOESM8_ESM.tif]

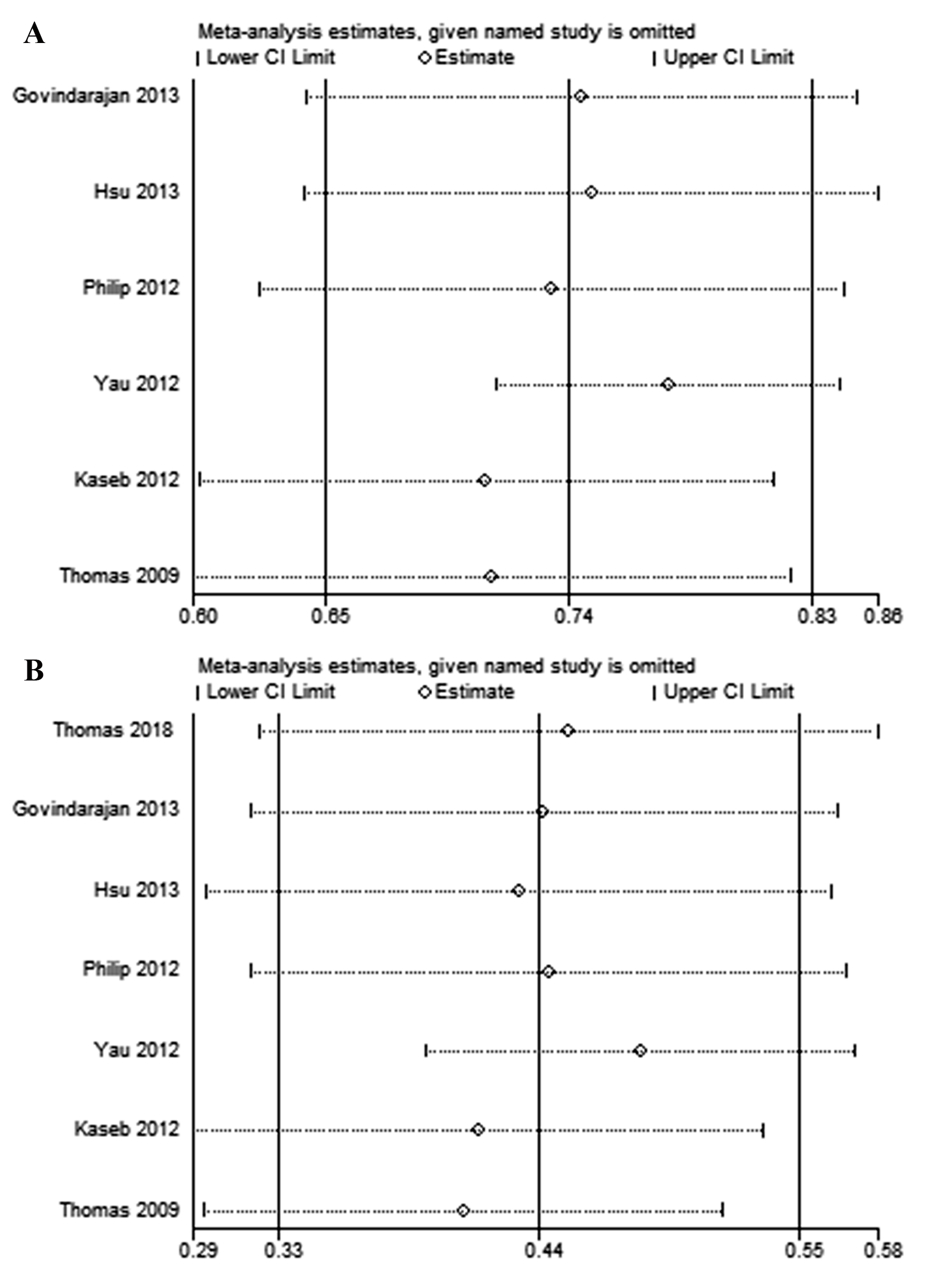

Supplement: Supplementary file 10 — Figure S4. Sensitivity analysis of OS-6 m (A) and OS-12 m (B). (TIF 14345 kb) [file 12885_2019_5487_MOESM10_ESM.tif]
