# Supplementary material for: Efficacy of bevacizumab combined with erlotinib for advanced hepatocellular carcinoma: a single-arm meta-analysis based on prospective studies
Source: BMC Cancer. 2019 Mar 28;19:276. doi: 10.1186/s12885-019-5487-6 (PMC6437948; doi:10.1186/s12885-019-5487-6)
Supplement: Supplementary file 5 — Table S4. Pooled disease control rate. (DCR) and modified DCR in the included advanced HCC patients. (DOCX 14 kb) [file 12885_2019_5487_MOESM5_ESM.docx]

**Table S4.** Pooled disease control rate (DCR) and modified DCR in advanced HCC patients included.

| **DCR (pre-deleted)** | | | | **DCR (post-deleted)** | | |
| --- | --- | --- | --- | --- | --- | --- |
| **Study** | **Mean** | | **95%CI** | **Study** | **Mean** | **95%CI** |
| Kaseb 2016 | 0.50 | (0.323,0.648) | | Kaseb 2016 | 0.50 | (0.323,0.617) |
| Hsu 2013 | 0.53 | (0.393,0.667) | | Hsu 2013 | 0.53 | (0.337,0.543) |
| Philip 2012 | 0.52 | (0.332 ,0.708) | | Philip 2012 | 0.52 | (0.262 ,0.690) |
| Kaseb 2012 | 0.80 | (0.698, 0.902) | | Thomas 2009 | 0.625 | (0.489,0.845) |
| Thomas 2009 | 0.625 | (0.489,0.845) | | **Total** | 0.545 | (0.469,0.0.668) |
| **Total** | 0.603 | (0.473,0.733) | |  | | |
| Overall (*I^2^*=76.4%, P=0.002); Egger’s test (P=0.076) | | | | Overall (*I^2^*=0, P=0.668); Egger’s test (P=0.917) | | |
