# Supplementary material for: Efficacy of bevacizumab combined with erlotinib for advanced hepatocellular carcinoma: a single-arm meta-analysis based on prospective studies
Source: BMC Cancer. 2019 Mar 28;19:276. doi: 10.1186/s12885-019-5487-6 (PMC6437948; doi:10.1186/s12885-019-5487-6)
Supplement: Supplementary file 7 — Table S5. Pooled progression-free survival rate. (PFS) and PFS-16w in the included advanced HCC patients. (DOCX 14 kb) [file 12885_2019_5487_MOESM7_ESM.docx]

**Table S5.** Pooled progression-free survival rate (PFS) and PFS-16w in advanced HCC patients included

| **PFS** | | | **PFS-16w** | | |
| --- | --- | --- | --- | --- | --- |
| **Study** | **Mean** | **95%CI** | **Study** | **Mean** | **95%CI** |
| Kaseb 2016 | 0.43 | (0.284,0.576) | Kaseb 2016 | 0.43 | (0.284,0.576) |
| Hsu 2013 | 0.353 | (0.222 ,0.484) | Hsu 2013 | 0.353 | (0.222 ,0.484) |
| Govindarajan 2013 | 0.28 | (0.088, 0.472) | Philip 2012 | 0.45 | (0.262,0.638) |
| Philip 2012 | 0.45 | (0.262,0.638) | Kaseb 2012 | 0.64 | (0.518, 0.762) |
| Kaseb 2012 | 0.64 | (0.518, 0.762) | Thomas 2009 | 0.625 | (0.475, 0.775) |
| Thomas 2009 | 0.625 | (0.475, 0.775) | **Total** | 0.502 | (0.382,0.622) |
| **Total** | 0.469 | (0.351, 0.588) | Overall (*I^2^*=70.6%, P=0.009); Egger’s test (P=0.732) | | |
| Overall (*I^2^*=72.9%, P=0.002); Egger’s test (P=0.353) | | |  |  |  |
