# Supplementary material for: Efficacy of bevacizumab combined with erlotinib for advanced hepatocellular carcinoma: a single-arm meta-analysis based on prospective studies
Source: BMC Cancer. 2019 Mar 28;19:276. doi: 10.1186/s12885-019-5487-6 (PMC6437948; doi:10.1186/s12885-019-5487-6)
Supplement: Supplementary file 9 — Table S6. Pooled overall survival rate at 6 months (OS-6 m) and modified OS-6 m in the included advanced HCC patients. (DOCX 14 kb) [file 12885_2019_5487_MOESM9_ESM.docx]

**Table S6.** Pooled overall survival rate at 6-month (OS-6m) and modified OS-6m in advanced HCC patients included.

| **OS-6m (pre-deleted)** | | | **OS-6m (post-deleted)** | | |
| --- | --- | --- | --- | --- | --- |
| **Study** | **Mean** | **95%CI** | **Study** | **Mean** | **95%CI** |
| Govindarajan 2013 | 0.687 | (0.489,0.885) | Govindarajan 2013 | 0.687 | (0.489,0.885) |
| Hsu 2013 | 0.691 | (0.564,0.818) | Hsu 2013 | 0.691 | (0.564,0.818) |
| Philip 2012 | 0.744 | (0.579,0.909) | Philip 2012 | 0.744 | (0.579 ,0.909) |
| Yau 2012 | 0.381 | (0.08, 0.682) | Kaseb 2012 | 0.836 | (0.742,0.930) |
| Kaseb 2012 | 0.836 | (0.742,0.930) | Thomas 2009 | 0.830 | 0.714, 0.946) |
| Thomas 2009 | 0.830 | 0.714, 0.946) | **Total** | 0.778 | (0.713,0.842) |
| **Total** | 0.740 | (0.648,0.832) | Overall (*I^2^*=19.5%, P=0.290); Egger’s test (P=0.161) | | |
| Overall (*I^2^*=56.8%, P=0.041); Egger’s test (P=0.022) | | |  |  |  |
