# Supplementary material for: Efficacy of bevacizumab combined with erlotinib for advanced hepatocellular carcinoma: a single-arm meta-analysis based on prospective studies
Source: BMC Cancer. 2019 Mar 28;19:276. doi: 10.1186/s12885-019-5487-6 (PMC6437948; doi:10.1186/s12885-019-5487-6)
Supplement: Supplementary file 11 — Table S7. Pooled overall survival rate at 12 months (OS-12 m) and modified OS-12 m in the included advanced HCC patients. (DOCX 14 kb) [file 12885_2019_5487_MOESM11_ESM.docx]

**Table S7.** Pooled overall survival rate at 12-month (OS-12m) and modified OS-12m in advanced HCC patients included.

| **OS-12m (pre-deleted)** | | | **OS-12m (post-deleted)** | | |
| --- | --- | --- | --- | --- | --- |
| **Study** | **Mean** | **95%CI** | **Study** | **Mean** | **95%CI** |
| Thomas 2018 | 0.370 | (0.270,0.370) | Thomas 2018 | 0.370 | (0.270,0.370) |
| Govindarajan 2013 | 0.416 | (0.205,0.627) | Govindarajan 2013 | 0.416 | (0.205,0.627) |
| Hsu 2013 | 0.475 | (0.338,0.612) | Hsu 2013 | 0.475 | (0.338,0.612) |
| Philip 2012 | 0.404 | (0.219 ,0.589) | Philip 2012 | 0.404 | (0.219 ,0.589) |
| Yau 2012 | 0.124 | (-0.08, 0.328) | Kaseb 2012 | 0.571 | (0.445,0.697) |
| Kaseb 2012 | 0.571 | (0.445,0.697) | **Total** | 0.449 | (0.368,0.530) |
| Thomas 2009 | 0.631 | (0.481, 0.781) | Overall (*I^2^*=37.9%, P=0.169); Egger’s test (P=0.844) | | |
| **Total** | 0.437 | (0.329,0.546) |  |  |  |
| Overall(*I^2^*=72.6%, P=0.001); Egger’s test (P=0.663) | | |  |  |  |
