# Supplementary material for: Efficacy of bevacizumab combined with erlotinib for advanced hepatocellular carcinoma: a single-arm meta-analysis based on prospective studies
Source: BMC Cancer. 2019 Mar 28;19:276. doi: 10.1186/s12885-019-5487-6 (PMC6437948; doi:10.1186/s12885-019-5487-6)
Supplement: Supplementary file 12 — Table S8. Pooled any-grade adverse effects (any-grade AEs) and grade 3–4 AEs in the included advanced HCC patients. (DOCX 15 kb) [file 12885_2019_5487_MOESM12_ESM.docx]

**Table S8.** Pooled Any-grade adverse effects (Any-grade AEs) and Grade 3-4 AEs in advanced HCC patients included.

|  | **Any-grade AEs** | | |  | **Grade 3–4 AEs** | | |
| --- | --- | --- | --- | --- | --- | --- | --- |
| **Toxicity** | **Study** | **N** | **Rate** |  | **Study** | **N** | **Rate** |
| Rash | 6 | 91 | 26.6% |  | 3 | 12 | 3.5% |
| Acne | 5 | 175 | 51.1% |  | 5 | 20 | 5.8% |
| Fatigue | 6 | 159 | 46.5% |  | 6 | 41 | 11.9% |
| Diarrhea | 8 | 186 | 54.4% |  | 7 | 31 | 9.0% |
| Hemorrhage | 6 | 126 | 36.8% |  | 6 | 18 | 5.3% |
| Hypertension | 7 | 65 | 19.0% |  | 6 | 23 | 6.7% |
| Nausea | 4 | 92 | 26.9% |  | 3 | 4 | 1.2% |
| Anemia | 5 | 18 | 5.3% |  | 3 | 7 | 2.0% |
| Hyperbilirubinemia | 3 | 35 | 10.2% |  | 4 | 6 | 1.7% |
| Anorexia | 4 | 117 | 34.2% |  | 4 | 4 | 1.2% |
