# Supplementary material for: Efficacy of bevacizumab combined with erlotinib for advanced hepatocellular carcinoma: a single-arm meta-analysis based on prospective studies
Source: BMC Cancer. 2019 Mar 28;19:276. doi: 10.1186/s12885-019-5487-6 (PMC6437948; doi:10.1186/s12885-019-5487-6)
Supplement: Supplementary file 13 — Table S9. Relatively common AEs of any grade in each included study. (DOCX 14 kb) [file 12885_2019_5487_MOESM13_ESM.docx]

**Table S9. Relatively common AEs of any grade in each study included**

| **Study** | **Rash** | **Acne** | **Fatigue** | **Diarrhea** | **Hemorrhage** | **Hypertension** | **Nausea** | **Anemia** | **Hyperbilirubinemia** | **Anorexia** |
| --- | --- | --- | --- | --- | --- | --- | --- | --- | --- | --- |
| Thomas 2018 | 16 | 27 | 33 | 41 | 0 | 21 | 24 | 0 | 13 | 28 |
| Kaseb 2016 | 3 | 45 | 29 | 26 | 21 | 8 | 17 | 6 | 14 | 21 |
| Govindarajan 2013 | NA | NA | NA | NA | 1 | NA | NA | NA | NA | NA |
| Hsu 2013 | 35 | 22 | 9 | 22 | 4 | 5 | 5 | 3 | 4 | 0 |
| Philip 2012 | 20 | 0 | 12 | 19 | 4 | 8 | 0 | 3 | 0 | 0 |
| Yau 2012 | 7 | 0 | 0 | 5 | 0 | 1 | 0 | 1 | 0 | 0 |
| Kaseb 2012 | 10 | 48 | 47 | 43 | 56 | 8 | 29 | 0 | 4 | 43 |
| Thomas 2009 | 0 | 33 | 29 | 30 | 40 | 14 | 17 | 5 | 0 | 25 |
