# Supplementary material for: Efficacy of bevacizumab combined with erlotinib for advanced hepatocellular carcinoma: a single-arm meta-analysis based on prospective studies
Source: BMC Cancer. 2019 Mar 28;19:276. doi: 10.1186/s12885-019-5487-6 (PMC6437948; doi:10.1186/s12885-019-5487-6)
Supplement: Supplementary file 14 — Table S10. Relatively common AEs of 3–4 grade in each included study. (DOCX 14 kb) [file 12885_2019_5487_MOESM14_ESM.docx]

**Table S10. Relatively common AEs of grade 3-4 in each study included**

| **Study** | **Rash** | **Acne** | **Fatigue** | **Diarrhea** | **Hemorrhage** | **Hypertension** | **Nausea** | **Anemia** | **Hyperbilirubinemia** | **Anorexia** |
| --- | --- | --- | --- | --- | --- | --- | --- | --- | --- | --- |
| Thomas 2018 | 0 | 9 | 3 | 4 | 0 | 4 | 0 | 0 | 1 | 2 |
| Kaseb 2016 | 0 | 5 | 6 | 4 | 4 | 1 | 1 | 3 | 1 | 1 |
| Govindarajan 2013 | 0 | 0 | 4 | 1 | 1 | 0 | 1 | 0 | 0 | 0 |
| Hsu 2013 | 0 | 5 | 0 | 3 | 2 | 1 | 0 | 1 | 3 | 0 |
| Philip 2012 | 6 | 0 | 2 | 4 | 1 | 1 | 0 | 0 | 0 | 0 |
| Yau 2012 | 1 | 0 | 0 | 1 | 1 | 0 | 2 | 0 | 0 | 0 |
| Kaseb 2012 | 0 | 4 | 18 | 10 | 6 | 8 | 0 | 0 | 1 | 1 |
| Thomas 2009 | 0 | 1 | 8 | 4 | 4 | 8 | 0 | 3 | 0 | 1 |
